# Evaluating the genetic effects of sex hormone traits on the development of mental traits: a polygenic score analysis and gene-environment-wide interaction study in UK Biobank cohort

**DOI:** 10.1186/s13041-020-00718-x

**Published:** 2021-01-06

**Authors:** Xiao Liang, ShiQiang Cheng, Jing Ye, XiaoMeng Chu, Yan Wen, Li Liu, Xin Qi, YuMeng Jia, Feng Zhang

**Affiliations:** 1 National Local Joint Engineering Research Center of Biodiagnostics and Biotherapy, The Second Affiliated Hospital of Xi’an Jiaotong University, Xi’an, China; 2 Key Laboratory of Trace Elements and Endemic Diseases, National Health Commission of the People’s Republic of China, School of Public Health, Health Science Center, Xi’an Jiaotong University, Xi’an, 71006 China

**Keywords:** Sex hormone traits, Gene-environment-wide interaction study (GEWIS), The frequency of alcohol consumption, Fluid intelligence

## Abstract

**Objective:**

To evaluate the genetic effects of sex hormone traits on the development of mental traits in middle-aged adults.

**Methods:**

The SNPs associated with sex hormone traits were derived from a two-stage genome-wide association study (GWAS). Four sex hormone traits were selected in the current study, including sex hormone-binding globulin (SHBG), testosterone, bioavailable testosterone and estradiol. The polygenic risk score (PRS) of sex hormone traits were calculated from individual-level genotype data of the United Kingdom (UK) Biobank cohort. We then used logistic and linear regression models to assess the associations between individual PRS of sex hormone traits and the frequency of alcohol consumption, anxiety, intelligence and so on. Finally, gene-environment-wide interaction study (GEWIS) was performed to detect novel candidate genes interacting with the sex hormone traits on the development of fluid intelligence and the frequency of smoking and alcohol consumption by PLINK2.0.

**Results:**

We observed positive association between SHBG and the frequency of alcohol consumption (b = 0.0101, *p* = 3.84 × 10^–11^) in middle-aged males and females. In addition, estradiol was positively associated with the frequency of alcohol consumption (b = 0.0128, *p* = 1.96 × 10^–8^) in middle-aged males. Moreover, bioavailable testosterone was associated with the fluid intelligence (b = − 0.0136, *p* = 5.74 × 10^–5^) in middle-aged females. Finally, GEWIS identified one significant loci, Tenascin R (TNR) (rs34633780, *p* = 3.45 × 10^–8^) interacting with total testosterone for fluid intelligence.

**Conclusion:**

Our study results support the genetic effects of sex hormone traits on the development of intelligence and the frequency of alcohol consumption in middle-aged adults in UK.

## Introduction

Mental disorders are highly prevalent and disabling globally, which lead to heavy burden on the health care system and society [1]. Based on the Global Burden of Disease, Injuries, and Risk Factors Study 2017 (GBD 2017), mental disorders consistently accounted for more than 14% of age-standardized years lived with disability for nearly 30 years [2]. It has been reported that 17.6% of adults suffered from a common mental disorder within the past 12 months and 29.2% across their lifetime [3]. Anxiety, alcohol use disorder and addiction are common mental disorders [3–5]. Interestingly, tobacco addiction is the most common co-occurring disorder among persons with serious mental illness [6]. The intelligence is linked to the risk of whole range of mental disorders [7].

Mental traits are multi-factorial, which are the result of multiple genetic and environmental factors that may interact in complicated ways to impact mental traits and disorders susceptibility. Addictions to alcohol and nicotine are heritable disorders [8]. The estimated heritability was 43% for tobacco usage and 19 ~ 29% for alcohol dependence [8]. Addictive disorder has a strong heritable component with an estimated heritability around 30 ~ 70% [9]. Sex differences in brain have known function to generate differences in the control of gonadotropic hormones, reproductive behavior or cognitive functions [10]. The sex differences in the dopamine response in the nucleus accumbens may affect the different vulnerability to addictive disorders in males and females [11]. Sex hormone activity strongly affects the individual’s behavior and the constitution of the brain [12].

Over past decade, there are robust evidences to support the relationships between hormones and mental disorders [13–15]. For instance, Lenz et al. [13] indicated that exposure to sex hormone in utero and during early development would contribute to the risk of alcohol addiction later in life. Interestingly, they also observed bidirectional relationship between the sex hormone axis and alcohol drinking behavior [13]. The association of hormone with alcohol intake was also supported by experimental animal study [16], which has identified that estradiol can stimulate alcohol consumption and aggression in male mice. In addition, it has been found that the change in hormone levels overtime and the ratio of progesterone to estradiol were the strongest hormonal predictors of smoking behavior [15]. Sex differences in anxiety-like behavior partially were affected by aged-related testosterone decline in male rats [17]. Stanikova et al. [18] suggested that higher free testosterone imbalance may mediate depression in overweight premenopausal women. However, limited efforts have been paid to explore the interactions effects between genetic factors and sex hormone traits for mental traits from sex-specific genetic perspective.

Polygenic risk score (PRS) is a sum of risk alleles, weighted by their effect size estimated from previous published genome-wide association study (GWAS). Utilizing identified susceptibility loci, PRS analysis can evaluate the effects of susceptible loci on disease risks and explore the genetic relationships between complex diseases and traits [9]. PRS has been applied in many studies on neuropsychiatric disorders [19, 20]. For instance, Jacqueline et al. [20] conducted PRS analysis to explore the possibility of overlapping genetic factors between smoking and the use of alcohol and cannabis. They found that PRS of cigarettes per day was associated with the number of glasses alcohol per week and cannabis initiation [20]. In another study, researchers found that the PRS of nicotine metabolism can predict nicotine metabolism biomarkers [21]. Additionally, Belsky et al. [22] observed that individuals with higher PRS were more likely to persist longer in smoking heavily and develop nicotine dependence more frequently. Recently, Katherine et al. [23] conducted a two-stage GWAS in 425,097 United Kingdom (UK) biobank study participants and identified 2571 genetic variant-sex hormone associations. Using the PRS of sex hormone traits as instrumental variables, we can calculate the PRS of sex hormone traits and explore the correlations between PRS of sex hormone traits and mental traits.

GWAS has great power to identify susceptibility genetic loci associated with mental disorders [24, 25]. However, the significant loci identified by GWAS are usually limited and functionally independent. Genetic effects are different between individuals due to gene-environmental (G × E) interactions, which resulted from individuals responding differently to environmental stimuli depending on their genotype [26]. Identifying G × E interactions would improve risk-assessment for complex diseases and reveal underlying biological pathways [26]. The gene-environment-wide interaction study (GEWIS) can estimate the effect of G × E interactions [27]. GEWIS can investigate the genetic interaction effect at a genome-wide scale, which can improve the ability of detecting genotype–phenotype associations missed in GWAS [27, 28].

In this study, we first calculated the PRSs of sex hormone traits in middle-aged adults in UK Biobank. Logistic and linear regression analyses were then performed to detect the associations between individual PRSs value of sex hormone traits and the phenotypic data of mental traits in UK Biobank. Finally, GEWIS was conducted to explore novel candidate genes interacting with sex hormone traits on the development of fluid intelligence and the frequency of alcohol consumption and smoking.

## Materials and methods

### UK Biobank samples and mental phenotypes

The phenotypic and genotypic data of this study were derived from UK Biobank health resource under UK Biobank application 46478, which was a population-based prospective cohort study. Between 2006 and 2010, UK Biobank collected 502,656 participants aged 40 and 69 at recruitment. UK Biobank cohort has collected a rich variety of phenotypic, health-related information on each participant, including physical and biological measurements, lifestyle indicators, imaging of the body and brain and genome-wide genotyping. Longitudinal follow-up for a wide range of health-related information are provided by linking health and medical records.

Several potential measures of smoking behavior were selected to define the phenotype of ever smoking. The UK Biobank data field of 20432 was described as ongoing behavioural or miscellanous addiction. Anxiety and depression were defined according to the previous study [29], which were based on the general anxiety disorder (GAD-7) and Patient Health Questionnaire (PHQ-9) [30, 31]. Fluid intelligence score was described as a simple unweighted sum of the number of correct answers given to the 13 fluid intelligence questions. The maximum number of reported past or current cigarettes (or pipes/cigars) consumed per day was used to define the frequency of smoking (UK Biobank data fields: 20116, 2887 and 3456). In addition, the frequency of alcohol consumption (UK Biobank data field: 20117) was defined as the sum of all alcoholic beverages per week. Ethical approval of UK Biobank study was granted by the National Health Service National Research Ethics Service (reference 11/NW/0382). The detailed definition of phenotypes are shown in Additional file 1.

### UK Biobank genotyping, imputation and quality control

A total of 488,377 middle-aged adults have genome-wide genotype data, which were assayed by two similar genotyping array. DNA was extracted from stored blood samples when participants visited to a UK Biobank assessment Centre. Genotyping was carried out by Affymetrix UK BiLEVE Axiom Array or the Affymetrix UK Biobank Axiom arrays (Santa Clara, CA, USA), which shared 95% of marker content. Imputation was conducted by IMPUTE4 (https://jmarchini.org/software/) to carry out in chunks of approximately 50,000 imputed markers with a 250 kb buffer region. Routine quality checks were carried throughout the process, including sample retrieval, DNA extraction and genotype calling. Statistical tests were performed to identify poor quality markers by checking for consistency of genotype calling across experimental factors, including batch effects, plate effects, departures from Hardy–Weinberg equilibrium (HWE), sex effects, array effects, and discordance across control replicates. Based on self-reported ethnicity (UK Biobank data field: 21000), the individuals were restricted to only “White British”. Finally, the participants who reported inconsistencies between self-reported gender or genetic gender, who were genotyped but not imputed, and who withdraw their consents, were excluded in the current study. Detailed description of array design, genotyping and quality control procedures can be found in the previous studies [32, 33].

### GWAS data of sex hormone traits

The SNPs associated with sex hormone traits were derived from a published GWAS [23]. Briefly, the published GWAS analyzed four sex hormone traits, including sex hormone-binding globulin (SHBG), testosterone, bioavailable testosterone and estradiol. Association test was conducted to account for cryptic population structure and relatedness by linear mixed models implemented in BOLT-LMM (v2.3.2). Genotypic data was derived from the ‘v3’ release of UK Biobank [32], which contained the full set of Haplotype Reference Consortium (HRC) and 1000 Genomes imputed variants. The SNPs with significant threshold of* p value *< 5 × 10^–8^ were selected to calculate PRSs. Detailed description of sample characteristics, array design, quality control and statistical analysis can be found in the previous study [23].

### PRS of sex hormone traits

Using the genotype data of UK Biobank cohort, PRS calculation was performed by using the PLINK’s “–score” command [34]. Briefly, PRS denotes the PRS of the sex hormone traits for the *ith* subjects, defined as $PRSi = \sum\nolimits_{n = 1}^{t} {\beta_{n} SNP_{ni} }$, where *n* (*n* = *1, 2, 3, …, t*) and *i* (*i* = *1, 2, 3, …, k*) denote the number of genetic markers and the sample size, respectively. *β*_*n*_ is the effect parameter of risk allele of the *nth* significant SNP related to sex hormone traits, which obtained from the published study. *SNP*_*ni*_ is the dosage (0 to 2) of the risk allele of the *nth* SNP for the *ith* subject. In addition, we have excluded Linkage Disequilibrium (LD) when calculating PRSs by using the command “–indep-pairwise” implemented in PLINK with parameters window size (500 kb), step size (5 variant ct) and r^2^ < 0.5.

### Statistical analysis

Four serum sex hormone traits, including SHBG, testosterone, bioavailable testosterone and estradiol, were analyzed both within and across sexes, with the exception of estradiol where analyses were performed only in men. Logistic regression model was performed to assess the associations between individual PRSs of sex hormone traits and ever smoking and ongoing behavioural or miscellanous addiction, respectively. Correspondingly, linear regression model was conducted to evaluate the correlations between individual PRSs of sex hormone traits and anxiety score, depression score, fluid intelligence score, and the frequency of alcohol consumption and smoking, respectively. 21 statistical tests (3 serum sex hormone traits × 7 mental traits) were analyzed by logistic and linear regression in total samples and females. The significant correlation was identified at *p* value < 2.38 × 10^–3^ (0.05/21) after Bonferroni correction. And 28 statistical tests (4 serum sex hormone traits × 7 mental traits) were analyzed by logistic and linear regression in males. The significant correlation was identified at *p* value < 1.79 × 10^–3^ (0.05/28) after Bonferroni correction. The regression analyses were conducted by R software (version 3.5.3). Sex, age, and 10 principle components of population structure were used as covariates in the regression model.

### GEWIS

Based on the results of regression model, GEWIS was performed to assess the interaction effects between genetic factors and sex hormone traits for fluid intelligence and the frequency of smoking per day and alcohol consumption per week in UK Biobank cohort. The GEWIS was conducted by PLINK2.0 [34, 35]. Letting D is the disease outcome variable, the penetrance models form is described as the following:

$${\text{logit}}[P(D = { 1}|G,E)] \, = \beta_{0} + \beta_{g} G + \beta_{e} E + \beta geGE$$

where G is genetic factor and E is the environmental factor [36]. In this study, the outcome variables were fluid intelligence score and the frequency of smoking per day and alcohol consumption per week. And the instrumental variables were the PRS of serum sex hormone traits. The HWE *p* value < 0.001 or minor allele frequencies (MAFs) < 0.01 or the SNPs with low call rates (< 0.90) were excluded in the current study for quality control. To avoid the influence of population stratification, cryptic relatedness and null model misspecifications on our results, we calculated the inflation factor of GEWIS. Significant interaction was identified at *p* value < 5.0 × 10^–8^ in this study. Rectangular Manhattan plot was generated using the “CMplot” R script (https://github.com/YinLiLin/R-CMplot).

## Result

A total of 7 significant associations were identified in this study, and the general characteristics of the subjects are presented in Table 1.

**Table1:** The associations between sex hormone traits and mental traits in males and females

		Number	Age ± Sd	*Beta*	*P* value
Total people	SHBG _ Frequency of alcohol consumption	388571	56.56 ± 8.07	0.0101	3.84 × 10^–11^
	Total T _ Frequency of alcohol consumption	388571	56.56 ± 8.07	0.0067	1.59 × 10^–5^
Males	Total T _ Frequency of smoking	185464	56.51 ± 8.22	− 0.0108	2.07 × 10^–6^
	SHBG_ Frequency of alcohol consumption	189153	56.84 ± 8.15	0.0090	8.18 × 10^–5^
	Estradiol _ Frequency of alcohol consumption	189153	56.84 ± 8.15	0.0128	1.96 × 10^–8^
Females	Bioavailable T _ Fluid intelligence	86777	56.46 ± 8.06	− 0.0136	5.74 × 10^–5^
	Total T _ Frequency of alcohol consumption	199167	56.29 ± 7.97	0.0102	4.55 × 10^–6^

Bioavailable testosterone (Bioavailable T); sex hormone-binding globulin (SHBG); Total testosterone (Total T)

### Associations between PRSs of sex hormone traits and mental traits in total middle-aged samples

Three sex hormone traits were analyzed in total middle-aged samples, including SHBG, total testosterone, and bioavailable testosterone. We observed positive associations between SHBG and the frequency of alcohol consumption (b = 0.0101, *p* = 3.84 × 10^–11^). In addition, total testosterone was positively associated with the frequency of alcohol consumption (b = 0.0067, *p* = 1.59 × 10^–5^). No significant association was found between bioavailable testosterone and mental traits in total middle-aged samples. The basic characteristics of study subjects and detailed information are presented in Additional file 2.

### Associations between PRSs of sex hormone traits and mental traits in middle-aged males

Briefly, four sex hormone traits were analyzed in middle-aged males, including SHBG, total testosterone, bioavailable testosterone and estradiol. Estradiol was positively associated with the frequency of alcohol consumption (b = 0.0128, *p* = 1.96 × 10^–8^). In addition, total testosterone was negatively associated with the frequency of smoking (b = − 0.0108, *p* = 2.07 × 10^–6^). SHBG was positively associated with the frequency of alcohol consumption (b = 0.0090, *p* = 8.18 × 10^–5^). No significant association was found between bioavailable testosterone and mental traits in middle-aged males. The basic characteristics of study subjects and detailed information are presented in Additional file 3.

### Associations between PRSs of sex hormone traits and mental traits in middle-aged females

Three sex hormone traits were analyzed in females, including SHBG, total testosterone, and bioavailable testosterone. Total testosterone was positively associated with the frequency of alcohol consumption (b = 0.0102, *p* = 4.55 × 10^–6^). We also observed negative association between bioavailable testosterone and fluid intelligence (b = − 0.0136, *p* = 5.74 × 10^–5^). No significant association was found between SHBG and mental traits in middle-aged females. The basic characteristics of study subjects and detailed information are presented in Additional file 4.

### GEWIS results

**Fig. 1:**
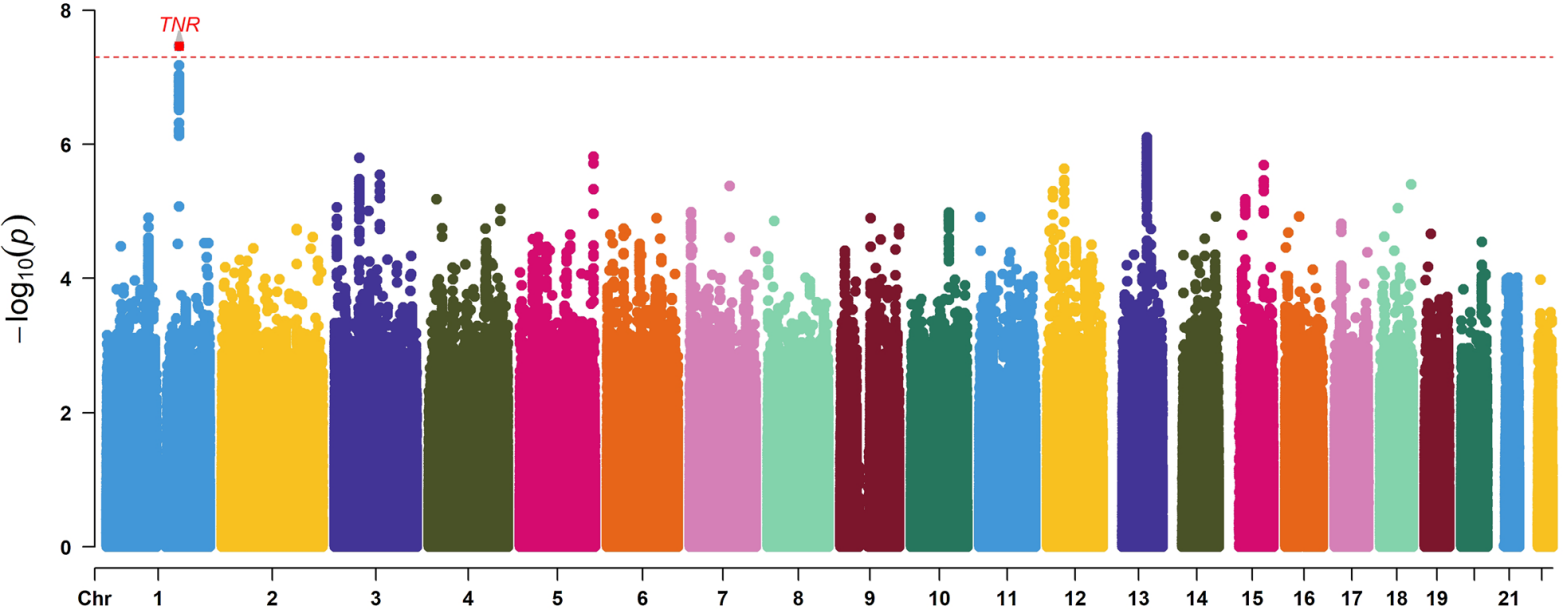
Genomic regions interacting with total testosterone for fluid intelligence. *Red plot represents the *p* < 5 × 10^−8^. The plots were generated using the “CMplot” R script (https://github.com/YinLiLin/R-CMplot)

**Fig. 2:**
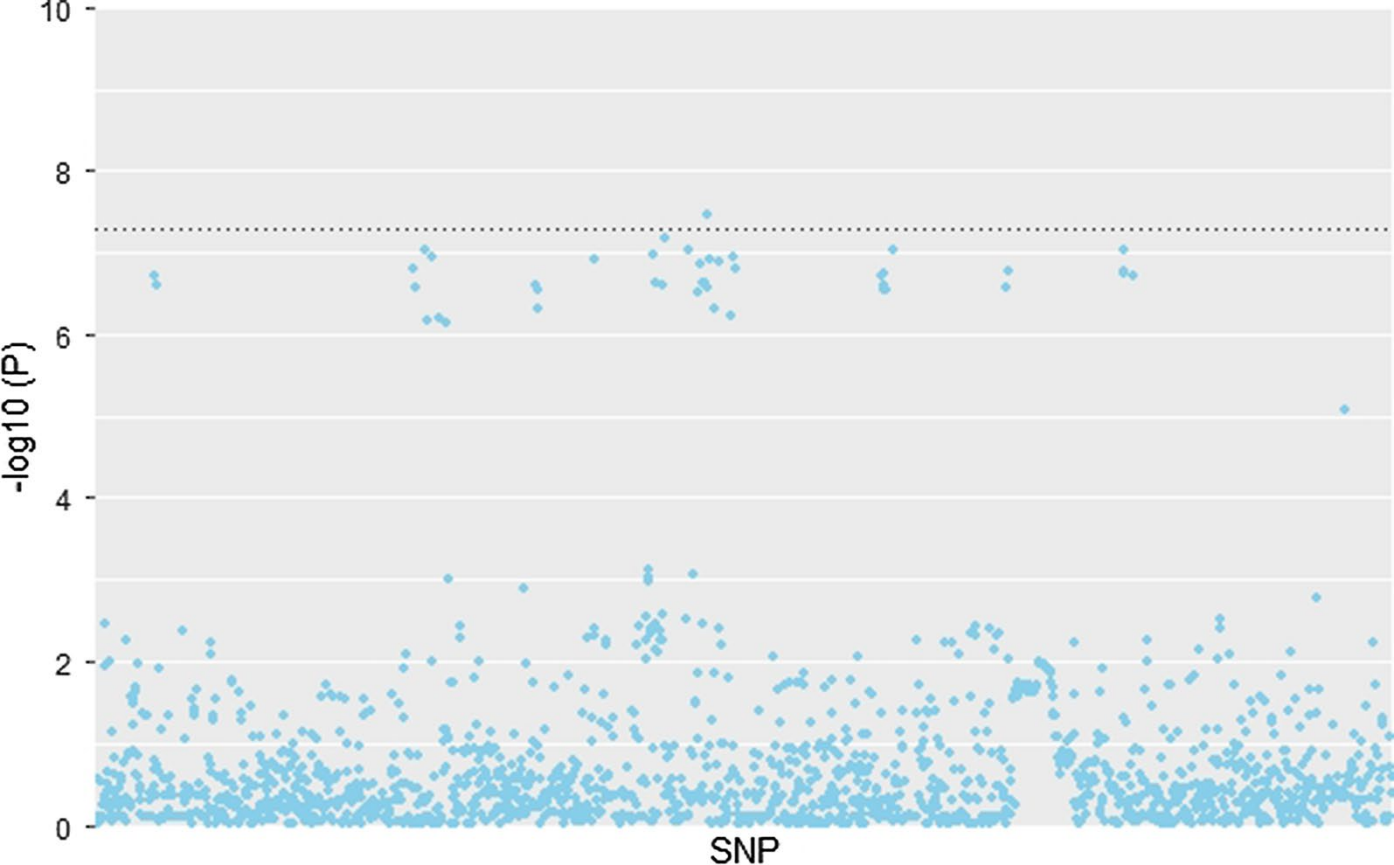
Scatter diagram of TNR. *The X-axis represents SNP, and the Y-axis represents −log10 p-values of each variant

The Rectangular Manhattan plot is shown in Fig. 1. The inflation factors was between 0.99 and 1.04 in this study, which indicates a low possibility of false-positive association resulting from population stratification and null model misspecifications. GEWIS identified one significant loci, Tenascin R (TNR) (rs34633780, *p* = 3.45 × 10^–8^) interacting with total testosterone for fluid intelligence in middle-aged adults. In addition, we identified several suggestive interaction signals (*p* < 5.00 × 10^−7^) for fluid intelligence, such as rs2301433 (*p* = 6.66 × 10^–8^) and rs61808374 (*p* = 9.52 × 10^–8^). Furthermore, we detected several loci interacting with total testosterone for the frequency of alcohol consumption, showing suggestive interaction signals (*p* < 5.00 × 10^−7^), such as rs116420771 (*p* = 5.95 × 10^–8^) and rs114191463 (*p* = 7.44 × 10^–8^). Finally, we found several loci interacting with total testosterone for the frequency of smoking, such as rs61841835 (*p* = 2.72 × 10^–7^) and rs61841834 (*p* = 2.79 × 10^–7^). The detailed information is presented in Table 2. The scatter diagram of TNR is shown in Fig. 2. All the suggestive SNPs in TNR are in LD (all pair-wise LD r^2^ > 0.787). The pair-wise LD r^2^ values of all suggestive SNPs are presented in Additional file 5.

**Table 2:** The summary of the genetic variant interacting with total testosterone for fluid intelligence (*p* < 5.0 × 10^–7^)

Chr	SNP	Effect alleles	Beta	*P* value	Gene
1	rs34633780	T	− 0.1051	3.45 × 10^–8^	TNR
1	rs2301433	A	− 0.1036	6.66 × 10^–8^	TNR
1	rs12742766	G	− 0.0986	9.29 × 10^–8^	TNR
1	rs743903	G	− 0.0986	9.35 × 10^–8^	TNR
1	rs2901906	C	− 0.0988	9.40 × 10^–8^	TNR
1	rs61808374	C	− 0.0984	9.52 × 10^–8^	TNR
1	rs2239818	A	− 0.0981	1.04 × 10^–7^	TNR
1	rs3766679	T	− 0.1045	1.11 × 10^–7^	TNR
1	rs3766678	T	− 0.1045	1.11 × 10^–7^	TNR
1	rs12753536	T	− 0.1044	1.15 × 10^–7^	TNR
1	rs34789755	G	− 0.1043	1.18 × 10^–7^	TNR
1	rs34842046	T	− 0.1042	1.22 × 10^–7^	TNR
1	rs1981473	C	− 0.1040	1.23 × 10^–7^	TNR
1	rs35627767	G	− 0.0973	1.31 × 10^–7^	TNR
1	rs34347370	A	− 0.1038	1.38 × 10^–7^	TNR
1	rs3795402	A	− 0.1034	1.57 × 10^–7^	TNR
1	rs12729778	C	− 0.1033	1.57 × 10^–7^	TNR
1	rs743902	T	− 0.0974	1.66 × 10^–7^	TNR
1	rs71645245	G	− 0.0974	1.66 × 10^–7^	TNR
1	rs61806420	A	− 0.0960	1.85 × 10^–7^	TNR
1	rs74888939	A	− 0.1037	1.86 × 10^–7^	TNR
1	rs74399607	A	− 0.1037	1.86 × 10^–7^	TNR
1	rs61806381	T	− 0.1022	1.90 × 10^–7^	TNR
1	rs10489319	C	− 0.1024	1.99 × 10^–7^	TNR
1	rs2282731	G	− 0.1015	2.27 × 10^–7^	TNR
1	rs34581198	C	− 0.0953	2.28 × 10^–7^	TNR
1	rs34442518	C	− 0.0950	2.42 × 10^–7^	TNR
1	rs61806384	G	− 0.0950	2.47 × 10^–7^	TNR
1	rs16848329	G	− 0.0950	2.48 × 10^–7^	TNR
1	rs10489320	G	− 0.1015	2.55 × 10^–7^	TNR
1	rs2301430	C	− 0.0949	2.57 × 10^–7^	TNR
1	rs12730963	C	− 0.0947	2.62 × 10^–7^	TNR
1	rs71645243	C	− 0.0947	2.75 × 10^–7^	TNR
1	rs34784860	G	− 0.1012	2.75 × 10^–7^	TNR
1	rs16848369	G	− 0.0945	2.83 × 10^–7^	TNR
1	rs61806423	A	− 0.0945	2.89 × 10^–7^	TNR
1	rs61806422	C	− 0.0945	2.90 × 10^–7^	TNR
1	rs34257437	G	− 0.0941	3.08 × 10^–7^	TNR
1	rs16848353	A	− 0.0933	4.77 × 10^–7^	TNR
1	rs35341067	T	− 0.0932	4.84 × 10^–7^	TNR

*Chr* chromosome, *SNP* single nucleotide polymorphism

## Discussion

Sex hormone supplementation has commonly effects on metabolic traits, sexual function and bone health [23]. Epidemiological study has indicated strong correlations between sex hormone supplements and health conditions [15]. However, the interactions effects between genetic factors and sex hormone traits for mental traits remain largely unknown now. Considering the effects of sex hormone traits on individual’s behavior and the constitution of the brain exhibiting fundamental differences between males and females, our study focuses on the potential impact of sex hormone traits on mental traits from sex-specific genetic perspective. We observed that sex hormone traits were associated with the frequency of alcohol consumption in middle-aged adults in UK. GEWIS further identified that the interaction of total testosterone with rs34633780 that mapped to the TNR gene can modulate the phenotype of fluid intelligence, possibly through influencing cognitive function.

TNR, a member of the tenascin family of neural extracellular matrix glycoproteins, primarily expressed in the central nervous system. This protein affects neural cell adhesion, neurite outgrowth and modulation of sodium channel function. It has been implicated that TNR was known to function in many neurological diseases [37, 38], such as attention deficit hyperactivity disorder (ADHD) [38] and neurodegenerative disorders [37]. David et al. [39] reported that an individual with a homozygous deletion of the TNR gene was associated with intellectual disability, supporting the role of TNR in brain development and cognition in humans. Moreover, Anna et al. [40] reported a case with intellectual disability had a 6.14 Mb duplication at 1q25.1–q25.2 by utilizing array comparative genomic hybridization (array CGH). Interestingly, the TNR gene located within this region (1q25.1–q25.2). Additionally, genome-wide association analysis found a novel association of ADHD with TNR gene [38]. In another study, researchers found that TNR deficiency would cause an early onset and nonprogressive neurodevelopmental disorder [41]. To the best of our knowledge, the study involving biological processes of TNR in fluid intelligence is still less. Although indirect, the “TNR-fluid intelligence” of GEWIS finding, combined with findings reported for other psychiatric disorders [37, 38, 41], supporting the evidence of TNR in the aetiology of psychiatric conditions.

Another significant finding of this study is the disclosure of the association between SHBG and the frequency of alcohol consumption in middle-aged males. SHBG, the major and specific binding protein for testosterone and estradiol, is known to regulate the bioavailability of sex steroids. Meanwhile, SHBG can assess bioavailable testosterone level [42]. Lee et al. [43] have reported that serum SHBG level is an independent predictive factor for extraprostatic extension of tumor in prostate cancer patients. Higher SHBG concentrations were observed in the premenopausal women who consumed alcohol [44], which consistent with our result in females. Interestingly, Markus et al. [45] demonstrated that serum SHBG level can be regulated by metabolic factors, including alcohol consumption and several drugs. Another study found that high concentrations of SHBG were consistently related to type II alcoholism [46]. However, Shiels et al. observed the paradoxical results. They found that decreased levels of SHBG were associated with increased alcohol consumption in United States men [47]. The inconsistent result of the association of alcohol with SHBG may be due to the samples size and different genetic background. The biological mechanism explanation for the correlation of SHBG with alcohol consumption is still unclear.

No association was observed between smoking status and SHBG in this study, which was consistent with previous study finding [47]. In addition, total testosterone was positively correlated with the frequency of alcohol consumption in middle-aged adults in UK. Interestingly, this result was also replicated in adolescent females [48]. For instance, Martin et al. [48] observed that females with higher levels of testosterone were more likely to be using alcohol currently. Although no direct effect of testosterone on alcohol consumption, researchers [49] have found that testosterone levels can predict future alcohol consumption. It has been identified that the frequency of alcohol consumption was positively associated with the concentrations of total testosterone among adult men [47]. In addition, high concentrations of total testosterone were shown to be associated with type II alcoholism [46]. Tina Kold et al. [50] suggested that alcohol consumption was associated with changes in testosterone levels among young men.

Estradiol played an important role in the establishment of sex differences in brain structure and function, which may act primary target for the investigation of sex-related differences in alcohol effects [51]. Our study results also supported that estradiol was positively associated with the frequency of alcohol consumption among middle-aged males. Population-based study in adolescent males found higher salivary estradiol level was associated with earlier onset and higher quantity of alcohol use [52]. Experimental animal study [16] suggested that estradiol can influence voluntary alcohol consumption and alcohol related behaviors in male mice, including aggression and depression. Most interestingly, these effects are strongly gender dependence [16]. Besides, previous study reported that alcohol consumption can affect gonadal hormone. For instance, Sarkola et al. [53] found that estradiol levels were increased after intake of alcohol among subjects who used oral contraceptives.

In addition, we observed that total testosterone was correlated with the frequency of smoking in middle-aged males. For instance, Ponholzer et al. observed that nicotine consumption was associated with serum levels of testosterone or free testosterone [54]. Another study demonstrated that current smokers of five or more cigarettes/day showed significantly higher levels of testosterone [55]. Significant increases in serum testosterone levels were observed in smokers group [56]. Similarly, Johan et al. observed that smoking men had 15% higher total testosterone levels compared with non-smoker [57], which was also demonstrated among current smokers [47]. Svartberg et al. found that smoking is an independent contributor to the variation of total testosterone and SHBG levels [58]. Interestingly, alcohol and tobacco have similar effects on plasma testosterone levels [59]. It has been identified that higher levels of alcohol and tobacco consumption were associated with higher levels of testosterone before and after alcohol withdrawal [59].

It is important to emphasize that our study has two limitations. First, all the samples were collected from UK Biobank cohort, aged between 40 and 69 at recruitment. Therefore, our findings should be carefully interpreted when applied to others ages and different genetic background populations. Second, the SNPs associated with sex hormone traits were derived from previous GWAS. The accuracy of our regression analyses may be influenced by the power of previous GWAS on the sex hormone traits. Further replication studies with other genetic background individuals and experimental studies are required to verify the results of this study.

In conclusion, the standardized collection of genotype and sex hormone supplementation data in UK Biobank give us an opportunity to access the interaction effect between sex hormone traits and genetic factors for mental traits. We observed correlations between sex hormone traits and the frequency of alcohol consumption and fluid intelligence in middle-aged adults. The most significant interaction effect was observed between total testosterone and TNR for fluid intelligence. Our study could provide novel insights into the impact of sex hormone traits on mental traits and highlight the importance of sex specific effects of sex hormone traits on mental traits.

The plots were generated using R script.

## Supplementary Information

**Additional file 1.** The detailed definition of mental phenotypes.

**Additional file 2.** . The associations between sex hormone traits and mental traits in males and females. **Additional file 2.1**: The associations between sex hormone traits and mental traits by logistic regression in males and females. **Additional file 2.2**: The associations between sex hormone traits and mental traits by linear regression in males and females.

**Additional file 3.** The associations between sex hormone traits and mental traits in males. **Additional file 3.1**: The associations between sex hormone traits and mental traits by logistic regression in males. **Additional file 3.2**: The associations between sex hormone traits and mental traits by linear regression in males.

**Additional file 4.** The associations between sex hormone traits and mental traits in females. **Additional file 4.1**: The associations between sex hormone traits and mental traits by logistic regression in females. **Additional file 4.2**: The associations between sex hormone traits and mental traits by linear regression in females.

**Additional file 5.** The pair-wise Linkage Disequilibrium (LD) r^2^ values of all suggestive SNPs in TNR gene.

## Data Availability

The UKB data are available through the UK Biobank Access Management System (https://www.ukbiobank.ac.uk/). We will return the derived data fields following UKB policy; in due course, they will be available through the UK Biobank Access Management System.
