# Supplementary material for: Evaluating the genetic effects of sex hormone traits on the development of mental traits: a polygenic score analysis and gene-environment-wide interaction study in UK Biobank cohort
Source: Mol Brain. 2021 Jan 6;14:3. doi: 10.1186/s13041-020-00718-x (PMC7788797; doi:10.1186/s13041-020-00718-x)
Supplement: Supplementary file 1 — Additional file 1. The detailed definition of mental phenotypes. [file 13041_2020_718_MOESM1_ESM.docx]

**Additional information 1**

***Definitions of criterion for ever smoking***

We selected several potential measures of smoking behavior in the UK Biobank. These include: 1) ever-tobacco smoker status; 2) former tobacco smoker status (among ever-tobacco smokers); 3) number of cigarettes per day, based on the previous study^1^. We coded ever smoker status as 1 if a respondent reported that they were a current or previous smoker, 0 if they reported never smoking and 0.5 if they reported only smoking less than once or twice per day.

***Definitions of criterion for ongoing behavioural or miscellanous addiction***

UK Biobank data field “20432” was defined as ongoing behavioural or miscellanous addiction. Participants were asked, “Are these addictions ongoing?”. Participants who answered “Yes” were classified as case group, and “No” were classified as control group.

***Definitions of criterion for anxiety score***

Anxiety was defined based on general anxiety disorder (GAD) question. GAD-7^2^ is a classification algorithm with a total score (0-21) used to screen for and measure anxiety severity, focusing on nine anxious symptoms and signs (as detailed below：Feeling nervous, anxious or on edge 20506, Not being able to stop or control worrying 20509, Worrying too much about different things 20520, Trouble relaxing 20515, Being so restless that it is hard to sit still 20516, Becoming easily annoyed or irritable 20505, Feeling afraid as if something awful might happen 20512. In order to meet the 0-3 score for each item of GAD, the 7 symptom scores (1-4) of our team UK were all reduced by 1 point.

***Definitions of criterion for depression score***

Depression was defined based on patient health question (PHQ). PHQ-9^2^ is a classification algorithm with a total score (0-27) used to screen for and measure depression severity, focusing on nine depressive symptoms and signs (as detailed below：Little interest or pleasure in doing things 20514, Feeling down, depressed, or hopeless 20510, Trouble sleeping 20517, Feeling tired 20519, Poor appetite or overeating 20511, Feeling bad about yourself 20507, Trouble concentrating 20508, Moving or speaking slowly or fidgety or restless 20518, Thoughts that you would be better off dead 20513). In order to meet the 0-3 score for each item of PHQ, the 9 symptom scores (1-4) of our team UK were all reduced by 1 point, which was then added up.

***Definitions of criterion for intelligence***

Intelligence (UK Biobank data field 20016) was defined according to a simple weighted sum of the correct answers to 13. Participants who did not answer all the questions within the prescribed 2 minutes scored zero for each question they did not attempt to answer.

***Definitions of criterion for frequency of alcohol consumption***

There are several phenotype options that measure drinking behavior in the UKB. After considering only phenotypes that cover the entire UKB sample, we were left with several to define alcohol per week. We used the sum of all alcoholic beverages per week as the weekly beverage phenotype for these respondents. Respondents who reported drinking less than once a week (one to three times a month, or only on special occasions) were asked how many different types of alcoholic beverages they consumed per month. For these subjects, we added up the total amount of alcohol consumed each month and divided it by 4 to get an approximate weekly amount of alcohol consumed. Those who never drank had a code of 0.

***Definitions of criterion for frequency of smoking***

We selected several potential measures of smoking behavior in the UKB. These include: 1) ever-tobacco smoker status; 2) former tobacco smoker status (among ever-tobacco smokers); 3) number of cigarettes per day, based on the previous study^1^. We coded cigarettes per day as 0 if ever-smoking status was also 0, otherwise, we used the maximum number of reported past or current cigarettes (or pipes/cigars) consumed per day.

## Appendix : Questionnaire wording and format

Introduction

Section A: present and past depression.

Section B: generalized anxiety disorder score.

Section C: smoking.

Section D: alcohol consumption.

| **Section A: present and past depression** | | | | |  |
| --- | --- | --- | --- | --- | --- |
| **PHQ score:** | | | | |  |
| 1. **20514** 2. **20510** 3. **20534** 4. **20519** 5. **20511** 6. **20507** 7. **20508** 8. **20518** 9. **20513** | | Over the last 2 weeks, how often have you been bothered by any of the following problems?  a. Little interest or pleasure in doing things  b. Feeling down, depressed, or hopeless  c. Trouble falling or staying asleep, or  sleeping too much  d. Feeling tired or having little energy  e. Poor appetite or overeating  f. Feeling bad about yourself or that you  are a failure or have let yourself or your  family down  g. Trouble concentrating on things, such  as reading the newspaper or watching  television  h. Moving or speaking so slowly that other  people could have noticed? Or the opposite — being so fidgety or restless that you have been moving around a lot more than usual  i. Thoughts that you would be better off  dead or of hurting yourself in some way | [Select one from the following for each of the statements]  - 01 Not at all  - 02 Several days  - 03 More than half the days  - 04 Nearly every day  - DA Prefer not to answer | |  |
| **section B: generalized anxiety disorder score** | | | | | |
| **GAD-7** | | | | | |
| 1. **20506** 2. **20509** 3. **20520** 4. **20515** 5. **20516** 6. **20505** 7. **20512** | Over the last 2 weeks, how often have you been bothered by any of the following problems?  a) Feeling nervous, anxious or on edge  b) Not being able to stop or control worrying  c) Worrying too much about different things  d) Trouble relaxing  e) Being so restless that it is hard to sit still  f) Becoming easily annoyed or irritable  g) Feeling afraid as if something awful might happen  [7 questions on one screen in grid] | | | [Select one from the following for each of the statements]  - 01 Not at all  - 02 Several days  - 03 More than half the days  - 04 Nearly every day  - DA Prefer not to answer | |
| **Section C: smoking** | | | |  |  |
| **20116** | The current/past smoking status of the participant | | | [Choose one of]  - 01 Previous  -02 Current  - 00 Never  - DA Prefer not to answer | |
| **2887** | About how many cigarettes did you smoke on average each day? (Count the total number of cigarettes (including both hand-rolled and  manufactured cigarettes if both were smoked)  For hand-rolled cigarettes:  - One ounce of tobacco makes about 30 cigarettes  - One gram of tobacco makes about 1 cigarette) | | | [Filing with numbers] | |
| **3456** | About how many cigarettes do you smoke on average each day? (Count the total number of cigarettes (including both hand-rolled and  manufactured cigarettes if both were smoked)  For hand-rolled cigarettes:  - One ounce of tobacco makes about 30 cigarettes  - One gram of tobacco makes about 1 cigarette) | | | [[Filing with numbers] | |
| **Section D: alcohol consumption** | | | |  |  |
| **20117** | The current/past alcohol drinking status of the participant | | | [Choose one of]  - 01 Previous  -02 Current  - 00 Never  - DA Prefer not to answer | |
| 1. **1568** 2. **1578** 3. **1588** 4. **1598** 5. **1608** 6. **5364** | In an average WEEK, how many glasses of wine would you drink? (There are six glasses in an average bottle)  a) RED wine  b) champagne plus white wine  c) beer plus cider  d) spirits  e) fortified wine  f) other alcoholic drinks | | | [Filing with numbers] | |
| 1. **4407** 2. **4418** 3. **4429** 4. **4440** 5. **4451** 6. **4462** | In an average MONTH, how many glasses of wine would you drink? (There are six glasses in an average bottle)  a) RED wine  b) champagne plus white wine  c) beer plus cider  d) spirits  e) fortified wine  f) other alcoholic drinks | | | [Filing with numbers] | |

**Reference**

1. Karlsson Linner, R. *et al.* Genome-wide association analyses of risk tolerance and risky behaviors in over 1 million individuals identify hundreds of loci and shared genetic influences. *Nat Genet* **51**, 245-257 (2019).

2. Kroenke, K., Spitzer, R.L., Williams, J.B.W. & L?we, B. The Patient Health Questionnaire Somatic, Anxiety, and Depressive Symptom Scales: a systematic review. **32**, 345-359.
