# Supplementary material for: Evaluating the genetic effects of sex hormone traits on the development of mental traits: a polygenic score analysis and gene-environment-wide interaction study in UK Biobank cohort
Source: Mol Brain. 2021 Jan 6;14:3. doi: 10.1186/s13041-020-00718-x (PMC7788797; doi:10.1186/s13041-020-00718-x)
Supplement: Supplementary file 2 — Additional file 2. . The associations between sex hormone traits and mental traits in males and females. Additional file 2.1: The associations between sex hormone traits and mental traits by logistic regression in males and females. Additional file 2.2: The associations between sex hormone traits and mental traits by linear regression in males and females. [file 13041_2020_718_MOESM2_ESM.docx]

**Additional file 2.1 The associations between sex hormone and mental disorders by logistic regression in total samples.**

|  | **Case** | **Control** | **Age ± Sd** | ***Beta*** | ***P* value** | **OR** | **OR_*P_5_*** | **OR_*P_95_*** |
| --- | --- | --- | --- | --- | --- | --- | --- | --- |
| **Bioavailable T _ Ever smoking** | 219436 | 265380 | 56.53±8.09 | -0.0054 | 6.38×10^-2^ | 0.99 | 0.99 | 1.00 |
| **Bioavailable T_Ongoing behavioural or miscellanous addiction** | 1044 | 1117 | 52.18±7.73 | -0.0383 | 3.77×10^-1^ | 0.96 | 0.90 | 1.03 |
| **SHBG _ Ever smoking** | 219436 | 265380 | 56.53±8.09 | 0.0084 | 4.27×10^-3^ | 1.01 | 1.00 | 1.01 |
| **SHBG _ Ongoing behavioural or miscellanous addiction** | 1044 | 1117 | 52.18±7.73 | 0.0193 | 6.56×10^-1^ | 1.02 | 0.95 | 1.09 |
| **Total T _ Ever smoking** | 219436 | 265380 | 56.53±8.09 | 0.0009 | 7.71×10^-1^ | 1.00 | 1.00 | 1.01 |
| **Total T _ Ongoing behavioural or miscellanous addiction** | 1044 | 1117 | 52.18±7.73 | 0.0972 | 2.75×10^-2^ | 1.10 | 1.03 | 1.19 |

*Note*：Bioavailable testosterone (Bioavailable T); sex hormone-binding globulin (SHBG); Total testosterone (Total T). Significant *P* values are in bold italics.

**Additional file 2.2 The associations between sex hormone and mental disorders by linear regression in total samples.**

|  | **Number** | **Women** | **Age ± Sd** | ***Beta*** | ***P* value** |
| --- | --- | --- | --- | --- | --- |
| **Bioavailable T _ Anxiety** | 151491 | 85339 | 55.90±7.74 | 0.0023 | 3.70×10^-1^ |
| **Bioavailable T _ Depression** | 150792 | 84955 | 55.91±7.74 | 0.0036 | 1.52×10^-1^ |
| **Bioavailable T _ Fluid intelligence** | 160121 | 86818 | 56.70±8.15 | -0.0044 | 6.68×10^-2^ |
| **Bioavailable T _ Frequency of alcohol consumption** | 388571 | 199265 | 56.56±8.07 | -0.0004 | 7.76×10^-1^ |
| **Bioavailable T _ Frequency of smoking** | 414294 | 228641 | 56.40±8.10 | 0.0015 | 3.32×10^-1^ |
| **SHBG _ Anxiety** | 151491 | 85339 | 55.90±7.74 | -0.0028 | 2.68×10^-1^ |
| **SHBG _ Depression** | 150792 | 84955 | 55.91±7.74 | -0.0001 | 9.69×10^-1^ |
| **SHBG _ Fluid intelligence** | 160121 | 86818 | 56.70±8.15 | 0.0058 | 1.65×10^-2^ |
| **SHBG _ Frequency of alcohol consumption** | 388571 | 199265 | 56.56±8.07 | 0.0101 | **3.84×10^-11^** |
| **SHBG _ Frequency of smoking** | 414294 | 228641 | 56.40±8.10 | 0.0018 | 2.27×10^-1^ |
| **Total T _ Anxiety** | 151491 | 85339 | 55.90±7.74 | -0.0018 | 4.95×10^-1^ |
| **Total T _ Depression** | 150792 | 84955 | 55.91±7.74 | 0.0005 | 8.55×10^-1^ |
| **Total T _ Fluid intelligence** | 160121 | 86818 | 56.70±8.15 | -0.0016 | 5.14×10^-1^ |
| **Total T _ Frequency of alcohol consumption** | 388571 | 199265 | 56.56±8.07 | 0.0067 | **1.59×10^-5^** |
| **Total T _ Frequency of smoking** | 414294 | 228641 | 56.40±8.10 | -0.0015 | 3.44×10^-1^ |

*Note*：Bioavailable testosterone (Bioavailable T); sex hormone-binding globulin (SHBG); Total testosterone (Total T). Significant *P* values are in bold italics.
