# Supplementary material for: Evaluating the genetic effects of sex hormone traits on the development of mental traits: a polygenic score analysis and gene-environment-wide interaction study in UK Biobank cohort
Source: Mol Brain. 2021 Jan 6;14:3. doi: 10.1186/s13041-020-00718-x (PMC7788797; doi:10.1186/s13041-020-00718-x)
Supplement: Supplementary file 3 — Additional file 3. The associations between sex hormone traits and mental traits in males. Additional file 3.1: The associations between sex hormone traits and mental traits by logistic regression in males. Additional file 3.2: The associations between sex hormone traits and mental traits by linear regression in males. [file 13041_2020_718_MOESM3_ESM.docx]

**Additional file 3.1 The associations between sex hormone and mental disorders by logistic regression in males.**

|  | **Case** | **Control** | **Age ± Sd** | ***Beta*** | ***P* value** | **OR** | **OR_*P*_5_** | **OR_*P*_95_** |
| --- | --- | --- | --- | --- | --- | --- | --- | --- |
| **Bioavailable T _ Ever smoking** | 113066 | 108603 | 56.74±8.19 | -0.0045 | 2.94×10^-1^ | 1.00 | 0.99 | 1.00 |
| **Bioavailable T _ Ongoing behavioural or miscellaneous addiction** | 643 | 746 | 52.65±7.83 | -0.0352 | 5.17×10^-1^ | 0.97 | 0.88 | 1.06 |
| **SHBG _ Ever smoking** | 113066 | 108603 | 56.74±8.19 | 0.0036 | 4.09×10^-1^ | 1.00 | 1.00 | 1.01 |
| **SHBG _ Ongoing behavioural or miscellanous addiction** | 643 | 746 | 52.65±7.83 | 0.0305 | 5.75×10^-1^ | 1.03 | 0.94 | 1.13 |
| **Total T _ Ever smoking** | 113066 | 108603 | 56.74±8.19 | -0.0099 | 2.13×10^-2^ | 0.99 | 0.98 | 1.00 |
| **Total T _ Ongoing behavioural or miscellaneous addiction** | 643 | 746 | 52.65±7.83 | 0.0431 | 4.27×10^-1^ | 1.04 | 0.95 | 1.14 |
| **Estradiol _ Ever smoking** | 113066 | 108603 | 56.74±8.19 | 0.0088 | 4.18×10^-2^ | 1.01 | 1.00 | 1.02 |
| **Estradiol _ Ongoing behavioural or miscellanous addiction** | 643 | 746 | 52.65±7.83 | -0.0430 | 4.30×10^-1^ | 0.96 | 0.88 | 1.05 |

*Note*：Bioavailable testosterone (Bioavailable T); sex hormone-binding globulin (SHBG); Total testosterone (Total T). Significant *P* values are in bold italics.

**Additional file 3.2 The associations between sex hormone and mental disorders by linear regression in males.**

|  | **Number** | **Age ± Sd** | ***Beta*** | ***P* value** |
| --- | --- | --- | --- | --- |
| **Bioavailable T _ Anxiety** | 66106 | 56.53±7.79 | 0.0013 | 7.32×10^-1^ |
| **Bioavailable T _ Depression** | 65793 | 56.54±7.79 | -0.0041 | 2.90×10^-1^ |
| **Bioavailable T _ Fluid intelligence** | 73236 | 56.99±8.25 | -0.0015 | 6.74×10^-1^ |
| **Bioavailable T _ Frequency of alcohol consumption** | 189153 | 56.84±8.15 | 0.0004 | 8.69×10^-1^ |
| **Bioavailable T _ Frequency of smoking** | 185464 | 56.51±8.22 | -0.0023 | 3.24×10^-1^ |
| **SHBG _ Anxiety** | 66106 | 56.53±7.79 | -0.0014 | 7.18×10^-1^ |
| **SHBG _ Depression** | 65793 | 56.54±7.79 | -0.0008 | 8.33×10^-1^ |
| **SHBG _ Fluid intelligence** | 73236 | 56.99±8.25 | 0.0055 | 1.23×10^-1^ |
| **SHBG _ Frequency of alcohol consumption** | 189153 | 56.84±8.15 | 0.0090 | **8.18×10^-5^** |
| **SHBG _ Frequency of** **smoking** | 185464 | 56.51±8.22 | -0.0001 | 9.79×10^-1^ |
| **Total T _ Anxiety** | 66106 | 56.53±7.79 | -0.0013 | 7.33×10^-1^ |
| **Total T _ Depression** | 65793 | 56.54±7.79 | -0.0053 | 1.73×10^-1^ |
| **Total T _ Fluid intelligence** | 73236 | 56.99±8.25 | 0.0027 | 4.52×10^-1^ |
| **Total T _ Frequency of alcohol consumption** | 189153 | 56.84±8.15 | 0.0050 | 2.69×10^-2^ |
| **Total T _ Frequency of smoking** | 185464 | 56.51±8.22 | -0.0108 | **2.07×10^-6^** |
| **Estradiol _ Anxiety** | 66106 | 56.53±7.79 | 0.0023 | 5.56×10^-1^ |
| **Estradiol _ Depression** | 65793 | 56.54±7.79 | 0.0014 | 7.10×10^-1^ |
| **Estradiol _ Fluid intelligence** | 73236 | 56.99±8.25 | 0.0084 | 1.90×10^-2^ |
| **Estradiol _ Frequency of alcohol consumption** | 189153 | 56.84±8.15 | 0.0128 | **1.96×10^-8^** |
| **Estradiol _ Frequency of smoking** | 185464 | 56.51±8.22 | 0.0054 | 1.77×10^-2^ |

*Note*：Bioavailable testosterone (Bioavailable T); sex hormone-binding globulin (SHBG); Total testosterone (Total T). Significant *P* values are in bold italics.
