# Supplementary material for: Evaluating the genetic effects of sex hormone traits on the development of mental traits: a polygenic score analysis and gene-environment-wide interaction study in UK Biobank cohort
Source: Mol Brain. 2021 Jan 6;14:3. doi: 10.1186/s13041-020-00718-x (PMC7788797; doi:10.1186/s13041-020-00718-x)
Supplement: Supplementary file 4 — Additional file 4. The associations between sex hormone traits and mental traits in females. Additional file 4.1: The associations between sex hormone traits and mental traits by logistic regression in females. Additional file 4.2: The associations between sex hormone traits and mental traits by linear regression in females. [file 13041_2020_718_MOESM4_ESM.docx]

**Additional file 4.1 The associations between sex hormone and mental disorders by logistic regression in females.**

|  | **Case** | **Control** | **Age ± Sd** | ***Beta*** | ***P* value** | **OR** | **OR_*P*_5_** | **OR_*P*_95_** |
| --- | --- | --- | --- | --- | --- | --- | --- | --- |
| **Bioavailable T _ Ever smoking** | 106168 | 156608 | 56.36±8.00 | -0.0077 | 6.04×10^-2^ | 0.99 | 0.99 | 1.00 |
| **Bioavailable T _ Ongoing behavioural or miscellanous addiction** | 400 | 369 | 51.36±7.47 | -0.0521 | 4.87×10^-1^ | 0.95 | 0.84 | 1.07 |
| **SHBG _ Ever smoking** | 106168 | 156608 | 56.36±8.00 | 0.0062 | 1.21×10^-1^ | 1.01 | 1.00 | 1.01 |
| **SHBG _ Ongoing behavioural or miscellanous addiction** | 400 | 369 | 51.36±7.47 | 0.0549 | 4.59×10^-1^ | 1.06 | 0.94 | 1.19 |
| **Total T _ Ever smoking** | 106168 | 156608 | 56.36±8.00 | 0.0038 | 3.46×10^-1^ | 1.00 | 1.00 | 1.01 |
| **Total T _ Ongoing behavioural or miscellanous addiction** | 400 | 369 | 51.36±7.47 | -0.0195 | 7.92×10^-1^ | 0.98 | 0.87 | 1.11 |

*Note*：Bioavailable testosterone (Bioavailable T); sex hormone-binding globulin (SHBG); Total testosterone (Total T). Significant *P* values are in bold italics.

**Additional file 4.2 The associations between sex hormone and mental disorders by linear regression in females.**

|  | **Number** | **Age ± Sd** | ***Beta*** | ***P* value** |
| --- | --- | --- | --- | --- |
| **Bioavailable T _ Anxiety** | 85295 | 55.41±7.65 | -0.0025 | 4.68×10^-1^ |
| **Bioavailable T _ Depression** | 84909 | 55.42±7.65 | 0.0047 | 1.75×10^-1^ |
| **Bioavailable T _ Fluid intelligence** | 86777 | 56.46±8.06 | -0.0136 | **5.74×10^-5^** |
| **Bioavailable T _ Frequency of alcohol consumption** | 199167 | 56.29±7.97 | 0.0008 | 7.09×10^-1^ |
| **Bioavailable T _ Frequency of** **smoking** | 228518 | 56.32±7.99 | 0.0010 | 6.45×10^-1^ |
| **SHBG _ Anxiety** | 85295 | 55.41±7.65 | -0.0071 | 3.79×10^-2^ |
| **SHBG _ Depression** | 84909 | 55.42±7.65 | 0.0002 | 9.54×10^-1^ |
| **SHBG _ Fluid intelligence** | 86777 | 56.46±8.06 | 0.0067 | 4.25×10^-2^ |
| **SHBG _ Frequency of alcohol consumption** | 199167 | 56.29±7.97 | 0.0067 | 2.60×10^-3^ |
| **SHBG _ Frequency of smoking** | 228518 | 56.32±7.99 | 0.0009 | 6.70×10^-1^ |
| **Total T _ Anxiety** | 85295 | 55.41±7.65 | -0.0016 | 6.37×10^-1^ |
| **Total T _ Depression** | 84909 | 55.42±7.65 | 0.0023 | 5.06×10^-1^ |
| **Total T _ Fluid intelligence** | 86777 | 56.46±8.06 | -0.0084 | 1.05×10^-2^ |
| **Total T _ Frequency of alcohol consumption** | 199167 | 56.29±7.97 | 0.0102 | **4.55×10^-6^** |
| **Total T _ Frequency of smoking** | 228518 | 56.32±7.99 | 0.0029 | 1.65×10^-1^ |

*Note*：Bioavailable testosterone (Bioavailable T); sex hormone-binding globulin (SHBG); Total testosterone (Total T). Significant *P* values are in bold italics.
